# Attention Deficits in a Comorbidity-Free Sample of Euthymic Pediatric Bipolar Disorder

**DOI:** 10.3389/fpsyt.2019.00148

**Published:** 2019-03-21

**Authors:** Nandini Banerjee, Shi-Kai Liu, Vinod K. Sinha, Meera Jayaswal, Pushpal Desarkar

**Affiliations:** ^1^Central Institute of Psychiatry, Ranchi, India; ^2^Centre for Addiction and Mental Health, Department of Psychiatry, University of Toronto, Toronto, ON, Canada; ^3^Department of Psychology, Ranchi University, Ranchi, India

**Keywords:** children, adolescents, pediatric bipolar disorder, attention, endophenotype

## Abstract

Attention deficits are considered one of the potential endophenotypic markers of Bipolar Disorder (BD). Pediatric bipolar disorder (PBD) likely has stronger genetic underpinnings than adult onset BD; therefore, demonstrating attention deficits in PBD can be both strategic and convincing in attesting their status as one of the potential endophenotypic markers of BD. However, unlike adult literature, uncertainty exists regarding the magnitude of attention deficits in PBD. In this regard, one key unresolved question is the potential impact of attention deficit hyperactivity disorder (ADHD). The main goal of the study was to examine attention deficits in a comorbidity-free sample of euthymic PBD patients. Thirty (21 boys, 9 girls) remitted PBD patients without co-morbidity and thirty age (<17 years), sex, handedness, and Full-Scale IQ matched control subjects were compared on performance on attention tasks. Working memory (WM), which might potentially confound with the attention task performances, was also examined. Compared to controls, PBD patients performed poorly on various tests of attention, but not on any WM tasks. Further, it was found that observed attention deficits were independent of residual mood symptoms, medication effect or illness characteristics. Such attention deficits in this comorbidity-free PBD sample further endorses its status as an endophenotypic marker of bipolar disorders and establishes continuity with deficits found in adult bipolar patients.

## Introduction

Impairments in attention have been demonstrated across symptomatic (1–3) and euthymic (4–7) phases in adult bipolar disorder (BD) patients and to variable degrees, in populations at higher risk for BD (4, 7–9). Further, naturalistic longitudinal study examining cognitive functioning in adult BD patients found that while cognitive function in BD varied significantly over time, a deficit in attention remained stable (5). Hence, attention deficits are considered one of the endophenotypic markers of BD (10). Pediatric bipolar disorder (PBD) likely has stronger genetic underpinnings than adult onset BD (9, 11), therefore, demonstrating attention deficits in PBD, especially in the euthymic phase, can be both strategic and convincing in attesting their status as one of the potential endophenotypic markers of BD. It also circumvents the methodological issue of course confounders in adult BD. Furthermore, given the potential detrimental impact of attention deficits on academic and social performance of children with BD (12, 13), understanding the nature and magnitude of attention deficits during the euthymic phase in this population has key therapeutic implications.

Despite such imports, unlike adult literature, uncertainty exists regarding the magnitude of attention deficits in PBD (14–16). Meta-analysis of studies comparing neurocognitive performance in PBD to healthy controls (17) revealed moderate differences in attention measures (*d* = 0.62); however, one striking finding was the substantial variability of effect sizes across studies on attentional measures (range 0.31–1.15). In this regard, one key unresolved question is the potential impact of psychiatric co-morbidities, notably attention deficit hyperactivity disorder (ADHD), which might have confounded the attentional performances (14, 18–22), with rare exceptions (23, 24). The other important point of consideration is the potential contribution of mood symptoms as most of these studies have been carried out in heterogeneous PBD samples with respect to symptom status, including children with hypomania and depression along with euthymic mood (14, 18, 19, 25).

In this consideration, current study is mainly aimed at specifically assessing attention deficits in euthymic PBD patients with co-morbidities carefully ruled out. Our secondary objective was to examine the potential impact of residual mood symptoms on neurocognitive measures. We anticipated that PBD patients will display significant attention deficits compared to controls.

## Materials and Methods

The current study enrolled 30 right-handed, euthymic [Young's Mania Rating Scale [YMRS] score <6] (26) and Hamilton Depression Rating Scale [HDRS] score <7] (27) PBD patients and 30 age-, sex- and IQ-matched healthy controls. All PBD patients were recruited from the Child and Adolescent Psychiatry outpatient clinic at the Central Institute of Psychiatry, India. In order to avoid unnecessary assessments on potentially ineligible participants, recruitment process in the study mainly involved receiving referrals from all 3 child and adolescent psychiatrists in the clinic who were made aware of the study eligibility criteria. One study author (NB) regularly visited the clinic and spoke with the psychiatrists and one study author (VKS) was one of the 3 psychiatrists working in the clinic. The protocol was approved by the Institute's Research Ethics Review Committee. Informed written consents were obtained from parents or legal guardians in accordance with the Declaration of Helsinki. The PBD patients were younger than 17 and met the ICD-10 DCR (28) criteria of BD. The diagnosis was further ascertained by interviewing the parents and child individually using the Kiddie-Schedule for Affective Disorders Present and Lifetime Version (K-SADS-PL) (29). All PBD subjects had been symptom free and on a stable medication regime at least for 2 months prior to the assessment.

Bipolar participants with the following conditions were excluded: co-morbid psychiatric disorders, especially ADHD; intellectual disability; unipolar depression and anxiety disorders, organic brain damages, harmful use of any substances over the past 6 months; severe or chronic co-morbid medical illness; and ECT treatment within 6 months. Participants with phobic disorders were included in the study. Participants taking corticosteroids or antihypertensive medications were excluded.

In total, we received 36 referrals from the clinic. Six participants were excluded after K-SADS-PL assessment, as 2 of them had previously undiagnosed anxiety disorders, 1 had undiagnosed ADHD and 3 did not meet the remission criteria. Although phobic disorders were included in the study inclusion criteria none of the included patients had any diagnosis of phobic disorder.

General intellectual ability (IQ) was measured by the Wechsler Intelligence Scale for Children- 3rd edition (WISC-III, UK) (30).

General attention tasks included the following: Trail Making Test, Part-A (TMT-A) (31) indicating psychomotor processing speed; Symbol Search Subtest of WISC-III (SS) as a pure test of information processing/perceptual speed (32); Coding Subtest (CS) of WISC-III as a time-paced test to assess psychomotor speed although better performance also involves good recall for the symbol-digit pairs. Sustained attention/vigilance was assessed by a computerized version of the visual Continuous Performance Test (CPT) (33) containing 350 characters (simple geometric designs) that were flashed on the screen at an interval of 750 ms. Subjects were required to press a bar whenever two identical stimuli appeared consecutively. The performance measures included number of correct responses, errors of omission, errors of commission, and response latency.

One important consideration here is the potential independence/interdependence between attention and working memory systems (34). In order to control for the potential confounding effect, working memory was assessed by Digit Span Backward (DS-B) Subtest of WISC-III (30) and the Spatial Working Memory (SWM) (Subtest of Cambridge Automated Neuropsychological Test Battery), which requires subject to update the working memory continually to avoid returning to previously searched locations. Scores are expressed as “between-search errors” (that is returning to boxes that previously have yielded tokens) and “strategy score” that is an index of how organized the search was. Higher scores on both measures indicate impaired performance. This study was approved by the Central Institute of Psychiatry Institute Research Ethics Board. All procedures related to this project are in accordance with the relevant guidelines and regulations.

### Statistical Analysis

The demographic characteristics and neurocognitive performances measures between PBD and control subjects were compared by either Chi- Square tests (categorical variables) or independent *t*-tests (continuous variables). To avoid multiple comparison and related type 1 error and also to control for the confounding effect of age and intelligence, a multivariate general linear model was devised with group (PBD vs. control) as between subject factors, neuropsychological measures as dependent variables and age, full scale IQ as covariates. After examining between-group differences, we used a set of *post-hoc* secondary analyses to examine the effect of medication, explore the relationship between those attention measures that showed significant group differences and clinical characteristics in PBD patients and finally examine the impact of sub-syndromal mood symptoms on the attention measures that showed significant group differences in PBD patients. In order to examine the effect of medication, three medication groups were created [monotherapy (*n* = 10), combination therapy (*n* = 20), and no medication, i.e., controls (*n* = 30)] and entered in the same multivariate general linear model described above as an additional “covariate.” Considering multicollinearity (since no control was taking medication), we checked the model and noticed that inclusion of medication subgroups as covariate did not alter the model and main study group effect size. Pillai's trace statistic was used to interpret all multivariate statistics. We also calculated effect size (partial eta-squared). The relationships between attention measures that showed significant differences between patients and controls and clinical characteristics in PBD patients were explored by Pearson's correlation coefficient. To further explore the impact of subsyndromal mood symptoms on sustained attention, performances between those PBD patients with significant (defined as having a score of 3 or more on either YMRS or HDRS) and negligible (<3 on both YMRS and HDRS) subsyndromal symptoms were compared by Mann–Whitney U. Statistical significance was set at *p* < 0.05.

## Results

There were no significant differences between PBD patients and controls in age (mean [SD]: 14.90 [1.37] vs. 14.96 [1.18], *p* = 0.84) and full IQ (94.20 [6.16] vs. 96.83 [6.57], *p* = 0.15). As well, there were no group difference in terms of education (*p* = 0.32), family income (*p* = 0.29), and location of residence (e.g., rural/urban) (*p* = 0.42). Clinically, the PBD patients had disease onset between 10 and 16 years, with an average duration of illness of about 1 year and <1 hospitalization during the disease course (Table 1). Among the PBD patients, nine (30%) had negligible subsyndromal mood symptoms. Ten patients were receiving monotherapy with either lithium (16.66%; *n* = 5) or sodium valproate (16.66%; *n* = 5) and 20 patients (66.66%) were taking combination therapy with a mood stabilizer plus an antipsychotic or combined mood stabilizers.

**Table 1:** Clinical characteristics of remitted pediatric bipolar patients (*N* = 30).

	**Mean ± SD**	**Range**
Age of onset (years)	13.73 ± 1.36	10–16
Duration of illness (months)	13.33 ± 8.74	5–36
Number of depressive episodes	0.33 ± 0.66	0–2
Number of months depressed	1.06 ± 4.37	0–24
Number of manic episodes	1.3 ± 0.65	1–4
Number of months manic	3.93 ± 2.44	1–10
Duration of remission (months)	6.66 ± 7.57	2–34
Number of hospitalizations	0.46 ± 0.50	0–1

We found significant group differences between euthymic PBD and controls on several measures of attention (Table 2), i.e., Trail Making Test (TMT-A), Symbol Search, Coding, and Continuous Performance Test (CPT), but not on verbal and visual working memory tasks. On TMT-A, PBD group took more time to complete the task than matched controls (*p* = 0.001). On Symbol Search (subtest of WISC-III), the group difference was significant on the measures of total correct (*p* = 0.026) and the difference between correct and incorrect responses (*p* = 0.032) or accuracy in performance, with the PBD group showing impairment on both the measures. With respect to Coding (subtest of WISC-III) too, group difference was significant (*p* = 0.003), with the PBD group earning significantly less scores than controls. Significant differences on sustained attention measures were also observed. On CPT, the remitted PBD group displayed significantly increased errors of omission (*p* = 0.04), and latency (response time) (*p* = 0.04). They also made fewer correct responses compared to controls (*p* = 0.01). The difference of performance on TMT-A time and Coding (subtest of WISC-III), which are indicators of psychomotor processing speed, was associated with a large effect size (Table 2). All other significant group differences on attention measures were associated with moderate effect size (Table 2). Thus, our sample size, i.e., 30 per group, was sufficient to detect published medium effect size (17) of attention deficits in the PBD group. The impairments were not likely caused by differences in response criteria, as the commission errors were comparable. In multivariate analysis, for PBD patients, medication did not impact the attention measures (Pillai's trace 1.66, F 1.11, *p* = 0.45).

**Table 2:** Comparison of neuropsychological measures of attention and working memory.

**Neuropsychological Variables**	**Patients (mean ± SD) (*N* = 30)**	**Healthy control (mean ± SD) (N=30)**	**F**	**Effect size (Partial η^**2**^)**	***p***
**ATTENTION**					
TMT-A time (s)	87.36 ± 43.70	52.83 ± 16.43	13.250	0.191	0.001^*^
Symbol search (Total)	22.43 ± 06.86	26.33 ± 06.72	3.735	0.063	0.058
Symbol search (Correct)	18.10 ± 06.03	22.23 ± 05.97	5.221	0.085	0.026^*^
Symbol search (Incorrect)	04.33 ± 02.77	04.10 ± 02.92	0.007	0.000	0.934
Symbol search (Correct-incorrect Dif.)	13.76 ± 06.39	18.13 ± 06.57	4.843	0.080	0.032^*^
Coding (Correct)	37.50 ± 08.90	45.60 ± 08.39	9.754	0.148	0.003^*^
**CONTINUOUS PERFORMANCE TASK (CPT)**					
CPT correct	29.50 ± 08.00	35.93 ± 08.52	6.320	0.101	0.015^*^
CPT error of omission	17.13 ± 07.72	11.63 ± 08.78	4.443	0.074	0.040^*^
CPT error of commission	18.03 ± 12.32	17.40 ± 14.58	0.027	0.000	0.870
CPT latency	00.60 ± 00.16	00.50 ± 00.16	4.261	0.071	0.044^*^
**WORKING MEMORY**					
Digit span backward	03.66 ± 01.09	03.96 ± 00.88	0.665	0.012	0.418
**SPATIAL WORKING MEMORY (CANTAB)**					
Between search error	36.10 ± 15.92	40.23 ± 20.31	1.319	0.023	0.256
Strategy	37.46 ± 05.87	36.30 ± 02.96	0.808	0.014	0.373

* *p < 0.05. CANTAB, Cambridge Automated Neuropsychological Test Battery*.

For PBD patients, those with significant subsyndromal mood symptoms (*n* = 21) had more CPT omission errors (18.90 ± 7.98) than those without (13.00 ± 5.4) (*p* = 0.04). There were no differences on TMT-A Time (*p* = 0.838), Symbol Search (Correct) (*p* = 0.683), Symbol Search (Correct-Incorrect) (*p* = 0.716), Coding (*p* = 0.717), CPT-correct (*p* = 0.415), or CPT latency (*p* = 0.213). Furthermore, there were no correlations between the attention measures and clinical/disease course characteristics in PBD patient either (Table 3), indicating relative independence of the neurocognitive performances to age of onset, number, and duration of symptomatic affective episodes.

**Table 3:** Relationship (Pearson's Correlation) between attention and clinical characteristics of remitted pediatric bipolar group (*N* = 30).

**Neuropsychological Measures**	**Age of onset (yrs)**	**Duration of illness (months)**	**No. of depressive episode**	**No. of months depressed**	**No. of manic episodes**	**No. of months manic**	**Duration of remission (months)**
**ATTENTION**							
TMT-A Time (s)	0.036	0.252	−0.003	−0.022	0.246	0.240	−0.227
Symbol search (Correct)	0.104	0.121	−0.130	−0.160	−0.247	−0.136	−0.146
Symbol search (Correct–Incorrect)	0.190	0.234	−0.177	−0.079	0.117	0.144	0.218
Coding (Correct)	0.315	0.057	0.047	−0.219	0.015	0.119	0.006
CPT correct	−0.126	−0.188	0.065	−0.154	−0.036	−0.302	0.099
CPT latency	0.011	0.008	0.155	−0.074	−0.016	−0.040	−0.033

## Discussion

To our knowledge, this current study is the likely the first one examining attention deficits in a comorbidity-free sample of euthymic PBD. Methodologically, psychiatric comorbidities that are likely to affect the attentional performances, such as ADHD (14, 18–22) were carefully ruled out and the impact of subsyndromal mood symptoms and medications were examined in the study. This type of study design is feasible in the Indian context as the described prevalence of ADHD in PBD is low, i.e., 4% (35).

In the present study, compared to controls, PBD patients performed poorly on various tests of attention, i.e., Trail Making Test (TMT-A), Symbol Search, Coding and Continuous Performance Test (CPT). The former three tests are all timed tests, which assess psychomotor speed while CPT assesses sustained attention. Doyle et al. (24) found similar deficits in Coding and CPT in their PBD sample and such deficits were independent of comorbid ADHD. Another study (36) reported deficits in attention on both TMT-A and CPT. Such deficits were found in both unmedicated symptomatic and medicated euthymic PBD patients.

In order to test our secondary objective, we conducted a *post-hoc* analysis to examine the impact of subsyndromal mood symptoms on cognitive performance. All group differences on attentional measures except error of omission on the CPT survived after controlling for subsyndromal mood symptoms. A previous study including adults with bipolar disorder reported similar findings, i.e., no deficit in error of omission after controlling for residual symptoms (37).

In our study, performance on attention tasks was not significantly affected by the use of mood stabilizers, either used as monotherapy or used in combination with another mood stabilizer or antipsychotic medication, when their impacts were examined statistically. This is consistent with the longitudinal follow up studies including adult (5) and PBD population (36). Furthermore, there is also some evidence that attentional measures may not be significantly altered by mood stabilizers or antipsychotic medications (38). A recent review reported the absence of any effect of lithium on attention in patients with bipolar disorder (39). Another recent study comparing cognitive effects of lithium and anticonvulsants, used either alone or as combination, in long-term stable bipolar patients found that performance on attentional measures are preserved in long-term stable bipolar patients taking these medications (40).

In summary, we obtained significant attention deficits in our PBD sample and found that such deficits were independent of working memory, medication effect or effect of subsyndromal mood symptoms. These observations further endorses the status of attention deficits as endophenotypic marker for PBD, as they are present in the very early stage of bipolar illness and they are not confounded by disease chronicity or medication use.

Functionally, the presence of attention impairments in PBD can have detrimental impacts on learning and education and socio-occupational functioning (12), as the acquisition of complex academic skills is predicated on intact attentional performance (13). Clinically, the course of attention deficits and their inter-correlation with clinical manifestations needs to be elucidated longitudinally. The improvements in neurocognitive performances might not parallel with clinical changes as demonstrated by slow modification of neural circuits over a longer time frame (41). Further, a longitudinal follow-up study involving PBD cohort revealed that improvements in cognition in the patients with PBD was comparable to that of the control subjects; however, cognition of patients with PBD still remained impaired relative to the controls. This finding indicates that illness disrupts cognitive development with potential lifelong implications for reduced functional ability (36). In our study, patients with PBD displayed attention deficits even though they were euthymic with medications. This finding underscores the need for identifying attention deficits as one important additional therapeutic target for the overall successful treatment of PBD patients.

In this study, we examined attention deficits in a comorbidity-free sample of PBD. However, our study is not without limitations. The PBD patients in our study were stable on medication, but they were not drug-free; therefore, potential contribution of medications still cannot be ruled out. Further, our study design was cross-sectional in nature; therefore, it was not possible to demonstrate longitudinal stability of attention deficits or clarify if such deficits were present pre-morbidly. Such studies with longitudinal design are needed to test the robustness of the status of attention deficits being endophenotypic markers of PBD. Finally, we acknowledge that our study sample size is small; therefore, the findings cannot be generalized without further investigation.

In conclusion, the current study provides further support in favor of attention deficits being a potential endophenotypic marker of PBD and establishes continuity with deficits found in adult bipolar patients. In the future, in depth longitudinal study of the neurophysiological and neuroanatomical correlates of attention deficits in a larger sample will not only shed lights on the etio-pathological process but will also help identify neural endophenotypes in Pediatric Bipolar Disorder. Further, our results underscore the need for specific interventions targeting attention deficits in PBD.

## Ethics Statement

The protocol was approved by the Institute's Research Ethics Review Committee. Informed consents were obtained from parents or legal guardians in accordance with the Declaration of Helsinki.

## Author Contributions

NB: data collection, assessments, analysis, interpretation, and manuscript preparation; S-KL: manuscript preparation and data analysis; VS: interpretation, manuscript preparation, study design; MJ: study design and manuscript preparation; PD: senior author, study design, interpretation, analysis, and manuscript preparation.

### Conflict of Interest Statement

The authors declare that the research was conducted in the absence of any commercial or financial relationships that could be construed as a potential conflict of interest.
